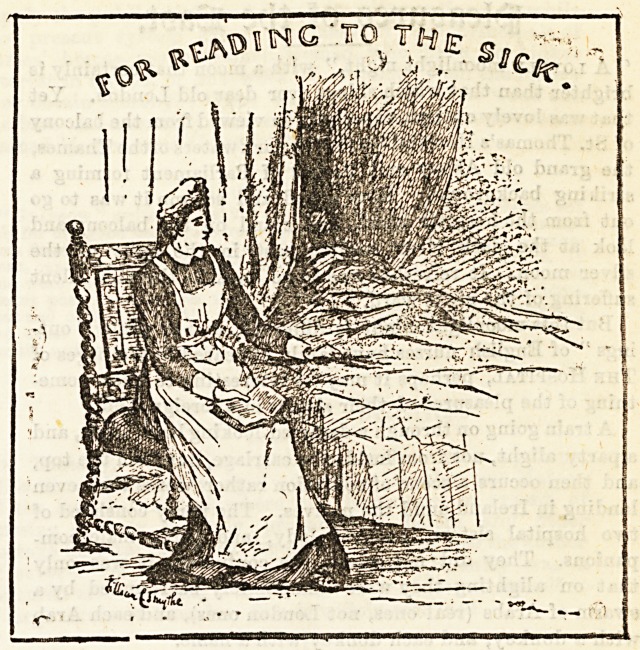# The Hospital Nursing Supplement

**Published:** 1891-09-26

**Authors:** 


### "The Hospital" Nursing Mirror.

Being the Extra Nursing Supplement of "The Hospital" Newspaper.

Contributions for this Supplement should be addressed to the Editor, The Hospital, 140, Strand, London, W.C., and should have the word "Nursing" plainly written in left-hand top corner of the envelope.

### En Passant.

NIGHTINGALE NURSES.—The report of the Nightingale School for 1890 is just out; there were 47 pupils admitted during the past year, and 32 completed their training. Miss Crossland, Dr. Bristowe, and Mr. Croft gave theoretical instruction to the probationers, and Miss Gordon reports that the probationers applied themselves diligently to their studies, and worked well in the wards. Miss Vincent reports that during the year 11 probationers finished their training at the Marylebone Infirmary. The Council call attention to the benefits offered by the Royal National Pension Fund for Nurses.

SHORT ITEMS.—Mrs. Rachel Norris leaves England early in October for the Riviera, where she will conduct a home for invalids.—A nurse writes and tells us of two cases of letters to nurses kept for weeks at a hospital instead of being immediately forwarded to their owners who were away at private cases. This is shameful, and if brought to the notice of the Postmaster-General would cause a scandal. —Nurse Epps, of Glasgow, writes to state that she was trained at the Deaconesses' Institution, not at the Infirmary, Tottenham.—With the sanction of the President, Princess Christian, Mrs. Margaret du Ane has been appointed to a vacant pension of £15 per annum in connection with the Trained Nurses' Annuity Fund.—St. John's Ambulance Association granted last year for nursing and first aid 28,000 certificates.—The Brassey Home has had 411 visitors in spite of the wet summer. There were two picnics last week, one to Bexhill-on-Sea and one to Ecclesbourne.—A nice-looking nurse, who says she was trained at Brownlow Hill, and who "borrows" money, is going about just now with false testimonials.

NURSING UNIFORMS.—The accusation lately brought by one of our correspondents against out-door uniform is not altogether unjust. Badly cut circular cloaks looking like extinguishers—but not extinguishing the big feet showing beneath—and small bonnets with stiff useless veils, are frequently seen about the streets. But also, be it remarked, that some very graceful and becoming uniforms can be seen, and we think it is the duty of those nurses who can afford it, to have their uniforms well-made, and their feet well shod. It does not cost more in the long run to get clothes from a good firm,—instance the very graceful cloaks made by Messrs. Debenham and Freebody, which are very light and full, and are waterproof. A Bloomsbury nurse lately told us that they last a life-time, and are as convenient as they are becoming. This firm supplies the uniform for the All Saints' Sisterhood, the St. John the Divine nurses, and others. Constantly we have had queries as to where nurses' uniforms could be best bought, and it was with real pleasure we found out at last that Messrs. Debenham and Freebody had arranged a special department—could make aprons or caps in a day, and supply galateas and merinos, and make gowns in two days. Once more we wish to insist that the veil is part of the uniform which ought to be very full and soft, and fall prettily; it should be long enough to be drawn about the face, and round the throat, on a cold day; on a windy day it should be drawn over one shoulder, and either twisted round the neck, or pinned to the front of the cloak. Being practical in all points, we give the following suggestions as to prices:—Cloaks can be had from 15s. 6d. up to 29s. 6d.; bonnets from 4s. 6d. to 10s. 6d.; galateas, 4d. or 6d. a yard.

THE NURSES' DIRECTORY.—It is a pity the R.B.N.A. cannot think of a plan of its own; it took up registration after the Hospitals Association had weighed the scheme and found it wanting, and it now proposes to get up a directory, when the training schools have been preparing a plan for the last six months. We know nothing of the directory the R.B.N.A. propose to produce (and remembering what a fiasco they made of their register we don't want to know anything), but the directory issued by the schools will, of course, be official, and should be supplied to the public on the 1st of January next. It will be compiled from the registers kept by the schools, so that the nurses now going about with forged certificates and testimonials will not be on it. Nurses receiving any papers referring to directories will do well to note if they refer to the *Official* Directory of Trained Nurses.

NURSES AND STUDENTS.—Some charming articles on nursing are now appearing in the *Guy's Hospital Gazette*, evidently from the pen of a student who combines childish conceit with sweet simplicity. Observe the following: "The success of hospital nursing, from the nurse's point of view, depends upon four great factors. (1) The attitude adopted by the students toward the nursing staff; (2) the attitude adopted by the ward Sister toward the nurses in her ward; (3) the point of view from which the nurse looks at her work; (4) the provisions made by the hospital authorities for housing and feeding the nurse, and for allowing her proper recreation and amusement." The food, housing of the nurses, etc., only comes fourthly, you see, but the student comes first! Here is a still more delicious note, which we commend to all the prim and proper Matrons: "There is no doubt that at a large number of hospitals the students do not try sufficiently to help the nurses in their training." Why should not the training of the nurses be handed over to these clever young students instead of left in the hands of sober old M.D.'s?

THE NURSE AND THE UNDERTAKER.—We have had much correspondence on this subject, and the comical as well as the serious side is coming forward. "I send you a 'Where is it,' sent me by an undertaker," writes one nurse, "and which I took as a delicate attention on his part. You will see that under U (for undertaker), and under B (for burial), and under C (for cremator), and under E (for embalmer), and under F (for funerals), he has written his address, and that a card with his terms is placed in the end. You will also see that I have never used the book for other addresses, for though it is handsomely got up, it gives me the shivers. Yet I must confess to having found it convenient to know the address which has been so persistently forced on my memory, for our duties cannot stop suddenly with death, when the whole house is sorrow-stricken, and perhaps no man present to see to the funeral arrangements." Some of the largest nursing institutions we find are "firm supporters" of this or that undertaker, and we regret to say that there can be no doubt that the heads of some of these institutions accept a commission. It is quite time that this "horrible alliance" was broken, and we are glad to say that the daily papers are ready to help us in this matter and give publicity to the names of all offenders should they persist in their evil ways after this warning.

### Lectures on Surgical Ward Work and Nursing.

By Alexander Miles, M.D. (Edin.), F.R.C.S.E.

Lecture XXXVI.—SAWS.

Saws naturally vary in size and shape, according to the particular part of the body in connection with which they are used.

(1) *The ordinary surgeon's saw* is used in dividing bones, for example, in an amputation of a limb. The blade is broad, the handle resembles that of a joiner's saw, and as a rule the back is made movable, so as not to interfere with the onward movement of the blade through a thick bone.

Fig. 1.

Fig. 3.

(2) *Metacarpal saws* (fig. 1) are of various patterns, some bow-shaped, others short and broad, and still others long and narrow. As the name implies, they are used to divide smaller bones, such as metacarpals or metatarsals.

Fig. 2.

(3) *Finger saw* (fig. 2) is still smaller, and is used to cut through the phalanges.

(4) *Butcher's saw* (fig. 3), called after the surgeon who intro-

Fig. 4.

duced it, consists of a narrow steel blade with fine teeth, set in a framework of metal in which it can be turned so as to work in any direction, and from which it may be readily detached. It is chiefly useful in excising joints, the narrow blade permitting of the bone being sawn in a direction away from the blood-vessels.

(5) *The bow-shaped saw* (fig. 1) possesses some, but not all, of the advantages of Butcher's.

(6) A *chain saw* (fig. 4) is sometimes used for dividing the neck of the femur, round which it is passed by the aid of a specially-curved needle.

(7) *Adam's saw* is used specially for the operation of subcutaneously dividing the neck of the femur which goes by the name of that surgeon. It consists of a long rounded stem, the terminal part only of which is serrated, and a large handle like that of an ordinary amputating saw.

Many other saws are in use, such as Annandale's jaw saw, Mr. Ewen's periosteotmy saw, saw for turbinated bones, and so on.

The illustrations are used by kind permission of Messrs. Maw, Son, and Thompson.

### Where to Go.

The holiday time draws to a close, and we have before us the long quiet hours of winter. It is too much the habit of nurses to narrow their lives, to let the world go by them, to leave papers and books unread and places unvisited. We cannot narrow our lives without narrowing our sympathies, and a nurse, above all women, needs to be able to comprehend the mental as well as the bodily trials of her patients, and she should also understand mental rest and recreation. Here are a few suggestions as to places where change of thought can be found. The Fabian Society give lectures at St. James's Hall at 8 p.m. on October 2nd and 16th, the first by Sydney Olivier on "Socialist Individualism," the second by Bernard Shaw on "The Difficulties of Anarchism," admission free. Miss Millington-Lathbury will lecture at the British Museum on October 8th, 15th, and 22nd, at 3 p.m., on "The Women of Greece." Ticket for the course 7s. 6d. The Swiney Lectures on Geology will be held at the Natural History Museum, South Kensington, on Mondays, Wednesdays, and Fridays at 3 p.m., commencing on October 5th, Professor Alleyn Nicholson, M.D., D.Sc., will deliver the course. Admission free. The Crystal Palace Saturday Concerts commence on October 10th at 3 p.m. A series of lessons on Invalid Cookery will be given during October at the Nurses' Club in Buckingham Street; particulars can be had from the Secretary. The Saturday Free Lectures in Toynbee Hall, Whitechapel, commence in October, when Mr. Barnett will lecture on "West and East." The two following lecturers will be Professor Nettleship and Mr. C. F. Tupper. The Working Men's College, Great Ormond Street, the Bow and Bromley Institute, the Charterhouse Institute, and others commence their autumn session in October, and particulars can be had from the Secretaries on sending stamped addressed envelopes. The Young Women's Christian Association, Regent Street, has arranged a series of evening classes on all subjects (including nursing) at nominal fees.

### Glasgow Royal Infirmary.

Mr. Henry Lamond, Secretary, writes: In consequence of certain representations by the nurses, communicated to the managers through the house physicians and house surgeons on Thursday last (previously to which date they had received no complaints), regarding the hours of duty and the food of the nurses, the managers appointed a special committee to investigate the statements made. The committee have since had several meetings, and have invited the nurses to come forward and state their complaints to them, and several of them have done so. The inquiry is still proceeding, and until the facts are fully and correctly ascertained, the managers trust that the public will suspend their judgment.

### To Would-be Nurses.

NEARLY every girl now-a-days wishes to become a nurse, but few seem to have the common sense to know how to set about it. The right road is to apply to the matron of some hospital which contains over 100 beds, or to answer advertisements for probationers.

But there are a few people to whom this course is useless; namely, those who are too young, who have imperfect health or strength, or who lack patience and courage. The lowest age at which nursing should be commenced is twenty for a children's hospital, and twenty-five for a general hospital, for girlhood is left behind after an insight into the sad scenes of hospital life, and no one has a right to throw away her youthful pleasures, any more than she has a right to try and retain those pleasures when the mature mind and body should be given to some earnest honest work. The would-be nurse, then, being old enough, and determined to take up work, should decide whether she will enter a big town hospital where the duties are heavy and the certificate valuable, or go to some small provincial hospital where the duties are light and the certificate of less account. It is more difficult to get into the big hospitals, and applicants often have to wait many months for a vacancy. We give particulars of a few nurse training schools, which may be taken as typical of similar institutions.

LONDON HOSPITAL, WHITECHAPEL.—Paying probationers are taken for not less than three months on payment of a guinea a week. Paid probationers (wages £10 and £15) have to sign for two years, and have to pass examinations to take their certificate. The hours on duty are twelve daily; each nurse has a separate bedroom. Age of admittance 25.

ST. THOMAS'S HOSPITAL, LAMBETH.—The Nightingale School in connection with this hospital takes probationers who pay a fee of £30 for their year's training; they sign for three years' service. Lectures and classes are given, but no certificates; the mere fact of being a Nightingale nurse is considered enough. Age 25. The time-table is as follows:—

| | | | |
|---|---|---|---|
| Rise ... ... ... | 6 a.m. | | |
| Breakfast ... ... | 6½ a.m. | Tea ... ... ... | 5 p.m. |
| Wards ... ... | 7 a.m. | Wards ... ... | 6 p.m. |
| Dinner ... | ¼ to 1 p.m. | Home ... ... | 8½ p.m. |
| Wards ... ... | 1.30 p.m. | Supper ... ... | 9 p.m. |
| Exercise | 11½ a.m. to ¼ to 1 p.m. or 3½ to 5 p.m. | Bed ... ... ... | 10 p.m. |

MARYLEBONE INFIRMARY, NOTTING HILL.—Salary for the first year £10, afterwards £20; must sign for three years. Age 22 to 32. Separate bedrooms, good lectures and classes.

BIRMINGHAM NEW INFIRMARY.—Fee for one year's training £20. Hours seven till nine, with two hours for recreation. Separate cubicles.

BRISTOL HOSPITAL FOR CHILDREN.—Two years course; certificate given; age 22; small premium.

SOHO HOSPITAL FOR WOMEN.—Course one year; certificate given. Premium 40 guineas. Age 22 to 30. Hours on duty twelve daily.

WINCHESTER COUNTY HOSPITAL.—Three years course; certificate given. No salary first year, £20 the second and third years. Hours seven to half-past eight, with two hours for exercise. Healthy situation outside the town.

WHITECHAPEL INFIRMARY.—One year's course; no salary given, and no premium required. Age 22 to 26.

For particulars of other nurse training schools see the *Hospital Annual*, price 3s. 6d., to be obtained at 140, Strand, London, W.C.

### Notice.

Two trained nurses are wanted for the Colonial Hospital, Perth, Western Australia. Passage out paid; salary £40 a year; charge of 18 beds each, quarters rough at present, as the Hospital is being re-organised. Further particulars can be had from the Crown Agents, Downing Street.

### A GARLAND.

WHO does not care for flowers? The rich man with his large hot-houses and conservatories, and the poor one with his window pots, are equally fond of these charms of nature. It is those, however, who delight in cultivating them, who watch the budding shoot and train the delicate branches, nipping off here a withered leaf, and there a straggling shoot, bringing all into a graceful shape and uniformity—it is they who are the true lovers of flowers.

God placed Adam in the Garden of Eden to dress it and to keep it, we read in the book of Genesis; and the same Creator has given each one of us the garden of the soul to keep in order, and we are to dress our hearts and minds and bring our very thoughts into union with His. Some people let their gardens run wild like the sluggard in the Proverbs.

"I passed by his garden, and saw the wild briar,
The thorn, and the thistle, grew broader and higher,"

says Dr. Watts in allusion to the same, and though it is spoken in reference to a pleasure ground, yet it is equally applicable to the mind and heart. We often see the spiritual sluggards, who love to take their ease and be at no trouble, let nettles and weeds of all sorts choke up and spoil their lives.

We will not compare the faults, sins, and follies of life with the thorns and thistles which overrun the earth, but will turn to a pleasanter subject, and try to find out what beautiful flowers we may raise to render our souls a little Paradise, that is a pleasure ground in the close neighbourhood of our King's palace. There are so many we hardly know which to choose out of the profusion, still, we cannot be far wrong in taking the rose of every shade, pink, white, red, and of every variety, and call them the love which suffereth long, and is kind to everybody. This is one of the best flowers to cultivate as being most pleasing to God and our fellow creatures. Then there is the lily, so pure and stately, sending forth a rich perfume; the humble violet, equally fragrant, giving and taking the blessing of the meek, sending out its most powerful odours when crushed; the carnation, bright and warm and spicy; the mignonette, with homely good qualities, surpassing our personal charms, possibly; the musk, so powerful to keep off evil scents. All these and many more should be growing freely in our garden, so that we may gather them hourly to make a beautiful garland day by day as the clock runs round its course.

There are a great many lovely blossoms which have no perfume in particular, such as the geranium and tulip, but they are worthy of culture since they are like good manners, which though nothing in themselves, yet when mingled with other flowers add a grace and charm to the nosegay. Let us try, then, to keep under our nettles and weeds and have always a garland of love, joy, peace, long suffering, meekness, gentleness, and purity ready for our Father's acceptance.

### Pleasures of the East.

"A LOVELY moonlight night," with a moon that certainly is brighter than that which shines over dear old London. Yet that was lovely enough, especially as viewed from the balcony of St. Thomas's Hospital—over the dark waters of the Thames, the grand old Abbey and Houses of Parliament forming a striking background. How grand and solemn it was to go out from the hospital ward, and stand on the balcony and look at the dark river rushing past in the light of the silver moon. It refreshed one to go back to the dim, silent suffering of the night ward.

But this moonlight night is different, and as some "outings" of English nurses have lately appeared in the pages of The Hospital, perhaps it may be interesting to know something of the pleasures of their sisters in a foreign land.

A train going on through a deserted-looking land, stops, and a party alight, not from inside the carriage, but from the top, and then occurs a scene of confusion rather worse than even landing in Ireland amid the natives. The party consisted of two hospital sisters, another lady, and three male companions. They did not make the confusion—it was only that on alighting they were immediately surrounded by a swarm of Arabs (real ones, not London ones), and each Arab with a donkey, and each donkey with a name.

"Him good, Mish-mish," cried one Arab; "take him lady."

"No, Billy Thompson better," cried another; and no shouting "eskut" (silence) could silence them, until at last all were mounted, and then with a sort of war-whoop off the donkeys started, each with a yelling Arab at its heels, and gaily we rode along a straight road until the scene changed to a sort of sandy desert with tall palm trees. Now and then a solitary tent appeared, the abode of wandering Bedouins. The tall trees cast deep shadows on the sand, but all is weird and lonely-looking and intensely silent, save for the merriness of the party.

"I shall not fall to-night, or at least be thrown," said one Sister—"not like last time."

"Why?" asked her companion.

"Because the patients in the women's ward say they will pray to the Virgin for me," was the reply. "I went in before going out. They like to see us English dressed for going out, and all admired my cap; and the Italians kindly volunteered me their prayers."

"Tell them to pray for me next time," replied her companion. "I believe I was thrown as well."

"Right you are. But let us get on." But lo! one donkey would not go. It was useless, like Mary in the rhyme, to beat it. It absolutely refused to go beyond a walking pace. And the only answer vouchsafed by the Arab boy when asked to make it go was—"God is patient, so should you be."

However, the scene was too lovely just now to want to hasten through it. A lake on one side shining like a silver sea—far on the other side the real sea, the rippling moon-lit sea of the Mediterranean, the tall trees with their crimson dates, all suggestive of the utmost calm. And then a little more and the party emerged on the banks of a canal. And how very un-English it looked; the lights in the hovels on the other side looking bright and fantastic, the silence only broken by the cry of the watchman, or rather men—as it seemed the shout of many—and occasionally a tall robed figure passed looking curiously at the English party as it passed. On the banks of the canal a halt was made for refreshments, which consisted of lemonade—the one who could speak Greek best being told off to provide it as the small restaurant on the canal bank was kept by a Greek.

"This *is* different to English life, certainly," remarked one, "which is best?"

"Oh, comparisons are odious," was the reply. "Just now this is *best*, but I believe a trip to Epping Forest or to Kew is considered quite a treat to some hospital nurses for a day. What would they think of this?"

"I wonder what one would think in England of being accosted by three or four different languages in a ward, and seeing each bed with a different nationality in it."

"I think we ought to be going home, or rather back. You know there is a day Sister sitting up till my return, and it is after eleven. Muskeen (poor thing), let us go;" and off the party started, going slowly as long as on the canal banks, but on emerging on the road breaking into a gallop—yes, a real gallop, and as only Eastern donkeys can gallop—six precious souls, and all agog.

We dashed through thick and thin, followed by our Arabs, and occasionally by starved-looking barking dogs. "This is a difference to last night," said one Sister to her companion; "a little more cheerful."

"What happened more than usual?"

"Only a man died, and all night two Italian priests exhorted him to repentance. It was rather weird and sad, the whole proceeding. How it still rings on my ear. 'Repento di tullo peccato, di mio vita passato.' Excuse the pronunciation, though; over and over again it was said, and then the chanted sort of prayer that was kept up."

"You see many strange sights, I expect, here abroad in hospitals?"

"Rather; very interesting, but occasionally confusing; and one longs for some good, honest English at times."

"Home, sweet Home," sang one, as down below us the town appeared in sight; the lights of the hospital, an imposing-looking building, appeared, and presently all alighted at the gate, which was opened by a sleepy porter, not using the best language, "only it was Greek," at being disturbed from his sleep. A tired day Sister appeared to greet them, and with many farewells the party separated, one Sister going to her well-earned rest, the other, refreshed by the outing, to stay at her work until the morning, only feeling she would be glad when seven a.m. came.

### Christmas Competitions.

Up to last Saturday we had already received for competition a dressing-gown from Nurse Ayrton, a flannel shirt from Miss S. Hale, and a pair of socks from Nurse Corner; for distribution, a pair of mittens from a Nurse, Heathfield; and 15 pairs of socks, six flannel petticoats and nine top petticoats from Mrs. William Black. The following are the prizes offered which will be awarded in books or money as the winners choose: (1) For the best pair of socks knitted by a nurse, 5s.; (2) for the best pair of socks knitted by any Hospital reader, 5s.; (3) for the best made flannel shirt, 10s.; (4) for the best made woman's blouse, 10s.; (5) for the best made flannel petticoat, 10s.; (6) for the best made and best shaped dressing gown for an invalid cut out and made by a nurse, 20s. It will be seen that No. 1 and 6 are reserved for nurses only. With regard to No. 6 we specially hope for many entries, and if we secure them we propose to give more than one prize. Flannelette is cheap, and light, and warm, and would, therefore, form the best material for the dressing gown. In judging, four marks are given for workmanship, four for shape, and two for general appearance; therefore, it is not wise to spend time on elaborate trimmings. Long seams may be done by machine. All garments whether sent in competition or not, will be distributed amongst the adult patients in hospital on Christmas day. The children are already well cared for by *Truth* and other papers, but the poor adult patients are seldom remembered. Yet a warm pair of socks or flannel petticoat to the man or woman recovering from pnuemonia or typhoid, is not only sure to cheer their Christmas day, but likely to help them to resist a relapse when they are discharged. Only a hospital Sister knows the feeling of weary despair which prevails, when a patient she has carefully nursed back to health, comes to say "Good bye" to her insufficiently clad, and perhaps going forth into a bitter east wind. We have known a Sister on such an occasion slip into her room and divest herself of her last flannel petticoat and give it away.

### A Bed for a Sick Nurse.

MANY an appeal have we urged on our nurse-readers lately, and promptly have they replied to one and all. But now we want to see if we have not thirty readers who are not nurses, but yet so far interested in nurses as to give a guinea a year in their cause. Constantly sad cases are brought before us of nurses who through illness are reduced to poverty, and who, sadly needing rest and sea-air, are unable to obtain the same. On February 7, we told how such a case was taken in free of charge at the Brassey Home, St. Leonards, and how the managers of the Home cared for the Nurse till she recovered, and then found her work. Since then we have constantly trespassed on the generosity of the Brassey Home, till our conscience begins to prick us; we feel it is not fair. To help us out of the difficulty, comes a letter from Nurse C. J. Bennett, enclosing a postal order for a guinea, and proposing that a bed be endowed for the use of sick nurses. The managers of the Home will put aside such a bed if they can secure thirty guineas a year, and two friends have already joined Nurse Bennett in subscribing a guinea—three out of the thirty before ever we began our appeal; now where are the other twenty-seven? The bed is absolutely for nurses recovering from illness, or very seedy, who have not the means to secure the nursing, rest, and sea-air they want, and who do not care to mingle with their own patients at an ordinary convalescent home. The bed will be in a separate little room looking out to sea, and already a small donation towards making the room pretty has been given. Under Sister Frost and Miss Holditch we are sure that small room will become a haven of rest; the interest they took in the scheme directly it was proposed is proof of this. Names and subscriptions will be received by Endowed Bed, The Hospital, 140, Strand, London, W.C. Gifts for beautifying the room should be sent to The Sister-in-Charge, Brassey Home, Ellenslea Road, St. Leonards-on-Sea.

### Everybody's Opinion.

[*Correspondence on all subjects is invited, but we cannot in any way be responsible for the opinions expressed by our correspondents. No communications can be entertained if the name and address of the correspondent is not given, or unless one side of the paper only be written on.*]

### NURSES AND RATS.

"AUNTIE" writes:—On Sunday afternoon as I was quietly reading and meditating on a number of the THE HOSPITAL, my peace was suddenly disturbed by the entrance of two merry girls shouting "Auntie, Auntie, look at this lovely creature." I looked, and softly enclosed in a pair of slender hands I saw a lovely little water-rat. Ought I to have screamed, dear Mr. Editor, as those young nurses did, and make myself generally foolish as they did also, when they were canoodling, as chronicled by you on August 15th? To an old-fashioned nurse, the nervousness of these young ladies (not to speak of the entire absence of mind shown by them in an emergency) points to a deficiency in their training not found in the pioneers of nursing such as Florence Nightingale and Florence Lees. Could a girl who screamed at a water-rat face quietly a delirious patient, or cope with the dangers of nursing a D.T. patient? Could they sit calmly by a fever patient in a lonely ward, and see more than one rat careering about the room? I fear not, and I trust, for the honour of our profession, there are only two such silly girls in it. Please, dear Mr. Editor, give more of such lovely sketches as the child story in last week's number, but no more adventures of hysterical girls. P.S.—HOSPITAL for 14th September just sent in, and yet another professedly river story. No one who knows the Thames or how to manage a boat could have written it.

### DRUNKEN NURSES.

"E." writes: As a nurse feeling a deep interest in the profession I am always grieved when any member falls short of the high moral standard which rightly belongs to those engaged in the noblest work a woman can be called to do. I quite agree with the Superintendent who writes that it is a disgrace that women such as she describes should be sent out to nurse the sick. For a long time I have noticed the great inability of principals and Superintendents of nurses' homes to form a proper judgment of the nurses they venture to supply the public with, and I fear it will continue so while the present system exists, as most of the principals of nurses' homes are untrained women, who have no higher interest in nurses, or the work of nursing, than the money they obtain thereby. What we want in order to weed out the base and unworthy nurses is associations of nurses under trained Matrons and Committees of Doctors.

### HOUSEKEEPING.

"GLASGOW" writes: I feel the subject of a Matron being chosen not only for her good certificate from a general hospital, but also for her diploma from the School of Cookery for cooking and household management, cannot be brought too often before the managers of hospitals. If this were required it would limit the number of applicants for each vacant post, causing less time and trouble in selecting a Superintendent and obtaining for the nurses a freedom from dyspepsia, anæmia, and other ailments from which they too often suffer from want of variety and palatable meals.

### Death in Our Ranks.

ON Tuesday, September 15th, suddenly, from syncope, Miss Kate M. Heanley, aged 40. Miss Heanley was Matron of the Boston Hospital, where she introduced a system of nursing entirely by lady probationers before any other cottage hospital had tried the plan. She was also the author of several very useful pamphlets. Miss Heanley was a cultured and charming woman, who had endeared herself to a large circle of friends. She is widely mourned.

### Notes and Queries.

### Queries.

(48) Recipes of cooling drinks for an invalid wanted, made without wine or spirits, and with as little acidity as possible.—*H. D.*

(49) Is there a special Nurses' Temperance Association with a pretty badge to be worn? Where is the office of the St. Barnabas Guild? Also of the Guild of St. Veronica?—*Nurse A.*

### Answers.

(47) Apply to the Mission House, Victoria Road, Worthing (12s. a-week); the Maghull Home, Liverpool (from 7s. a-week); St. Lucy's Home, Hare Lane, Gloucester; or to Miss Lobb, Convalescent Home, Lambourne, Essex.

*The Maligned District Nurse.*—Your letter arrived twenty-four hours after we had gone to press. We advise you to let the matter drop. If we publish your letter the institute will probably commence legal proceedings against you.

*Alpha.*—Dispensing can be learnt for ten guineas for the full course at the Middlesex College of Pharmacy, 40, Charlotte Street, Portland Place, W. At Warneford Hospital, Leamington, for ten guineas, for a six months' course. You have to attend and pass an examination at the Society of Apothecaries (fee, two guineas). The examination will be as follows: In translating prescriptions; in the British Pharmacopœia; in Materia Medica and Botany; in Pharmaceutical Chemistry; in Pharmacy and Dispensing. Such Latin as is necessary will be taught you at the College. You can also get lessons at the New Hospital for Women, the Zenana Medical College, and other places.

*Nurse C.*—It is usual to give a monthly nurse who has been engaged, but who is not needed, the half of her fee. Any medical man will uphold you in this demand.

*Nurse E. R.*—Thanks for your sensible letter, but this question of water-beds must be dropt now, we are so short of space.

*Midwife.*—Southall's accouchement sheets and sanitary towels; we thought every nurse knew of these valuable inventions.

*H. D.*—Get "Diet for the Sick," by Dr. Ridge. Published by Churchill. Price 1s. 6d.

*Old Indian.*—Next week. Thank you.

*Nurse Catherine.*—For 15 guineas you can get three months' training at the Metropolitan District Nursing Association, Bloomsbury Square, W.C.

*Gainsboro'.*—See article "Would-be Nurses," on page cli.

*Nurse Edith.*—Miss Parmenter, Russell's Farm, Braintree, Essex.

*Leavesden.*—Dr. Walmsley's pamphlet is published at the *Observer* office, Watford.

### Wants and Workers.

*Jaffray Surburban Hospital, Gravelly Hill, Birmingham.*—The Matron would be most grateful for red flannel bed jackets, cot counterpanes, toys, or games, and children's left-off clothing, and would pay carriage on any parcels sent for the patients.

*The Cottage Hospital, Valencia Island, co. Kerry, Ireland.*—An appeal of an urgent nature reaches us from the Committee of the above excellent little institution. The funds, owing partly to the great increase in the price of fuel and food during last winter, and partly from the falling off of subscribers, are at the very lowest ebb; also, through an increase of very expensive cases requiring expensive diet. Funds are most earnestly applied for, as, unless the new year, commencing October, is begun out of debt, this most valuable help to the poor in times of illness must be closed.

*Sister Garrard* asks any one interested in promoting the happiness of invalid children to send old perambulators for the use of the babies at the Convalescent Home, Lambourne, Essex.

### Content to Follow.

*(Concluded.)*

Not for one moment did Miss Rayton regret that she had taken this small-pox case.

"I am glad George does not know," she thought as she took the tumbled clothes from the bed, and arranged them neatly and comfortably over the poor disfigured form which lay there. "He would not like me to be here."

Then a sudden sharp pang, as the thought flashed before her that the merciless disease, which had already seized on one life for its prey, would probably claim her for its next victim.

There was no selfish thought for herself, only a moment of bitter grief to think of the trouble her act might bring on one whom she loved far better than her own life; but almost at the same time came the comforting consciousness that had he known all, he could not have wished her to do otherwise than what she felt it her duty to do.

"If I proved false to what I know is right, I should not be worthy of him," she thought, and her heart grew light once more.

The long, weary night passed slowly by. No sound of life was heard in that lonely, deserted house, but the low moans of the sufferer, as he moved restlessly from side to side.

The restlessness gradually gave way, and passed into a heavy, exhausted sleep, from which there seemed faint hope that he would ever rouse again.

"He does not know—he will never know—that anyone is with him," she thought.

"I wonder who he is, and who his friends are. Are they waiting anxiously for him at this moment? How dreadful to die so far away from all who care for you."

She sat still and listened to the distant chimes of the church clock striking the quarters, at what seemed to the watcher such interminable intervals, hardly taking her eyes from her patient, so anxious to note the slightest change. Would he never speak again? Had she given up so much for no purpose? Would it make no difference to the dying man that she had chosen that post by his bedside at the risk of losing all that made the future look bright and beautiful.

But if he never knew it, what then? It was still true that her place was there, that duty's voice had called her to that spot, and had not made promises that this or that reward should follow. While she was chiding herself for allowing these unworthy thoughts to take possession of her in this way, the man's eyes suddenly opened, and he raised his head quickly from the pillow. His sunken eyes glanced round the bare and wretched room, the cold moonlight fell in patches on the floor, while the chill night breezes coming fitfully through the open window made the flickering candle cast strange shadows on the ceiling and walls.

The expression of utter hopeless misery, depicted on that face, when the poor creature fancied himself left to die alone, will never be forgotten while Mary lives.

His terrified look, sought in an agony of fear for some sight or sound of human life, and it fell suddenly on the figure of a woman seated by his bed-side, her hand already on his, to assure him of her presence, while her low voice, close to his ear, broke the dreadful silence. "Do not be frightened, I am here, I am not going away."

He fixed his eyes on her.

Who was this? who like a guardian angel had appeared in his great loneliness, and who kept repeating the comforting words,

"I am not going away," until his weak and confused brain grasped the fact that he had not been left to die alone; but that somehow, he could not think collectedly enough to reason how, help and comfort were close at hand, and the dread was passed.

He sank back exhausted, but the fear was gone.

He looked at her to make sure she was not a vision, which might vanish, then smiled to feel still the pressure of a real warm hand upon his own.

"I am happy now," he said faintly. "I don't mind anything if you will stay. You said you would stay. You are not going?"

"You need not be afraid; I am not going away."

He tried to murmur some thanks. She stooped over him to catch what he said, and to speak some words of consolation and hope, but she only heard the whispered, "You will stay?" as he sank into that dim borderland of life, where no sound from the world would reach him more.

Only by the heavy breathing did she know that he still lingered, while the first gleam of dawn broke through the small window which looked eastward.

> "But when the morn came dim and sad,
> And chill with early showers,
> His quiet eyelids closed. He had
> Another morn than ours."

Mary had hardly realized that her task was over when Dr. Coles drove hurriedly to the door, and quickly entered the room.

"Ah! I was afraid I should be too late," he said. "I missed the road somehow coming back, and went miles out of the right way. I can't tell you how thankful I felt all the time that the poor fellow was in your charge. May you have your reward, Miss Rayton. He could not repay you, nor can I; but you will not lose it nevertheless."

And Mary knew, though she could not have put her feelings into words, that a rich reward was already hers in the consciousness that her presence that night in that miserable room had made the difference to one poor soul between the darkness of despair and the light of hope, between the utter desolation of one forsaken and the gratitude of one helped and comforted. She, taking no thought of pleasure, had found a pleasure which would abide with her through life.

> "All worldly joys go lesse
> Is the one joy of doing kindnesses."

. . . . . . . . . .

Not many months later, Dr. Coles received a letter from India, in which Mary (Miss Rayton no longer) described at some length her new home, and added "I told George all about the small pox case, and I am glad that he does not mind my having nursed it. At any rate, he does not seem very angry *now*."

### Amusements and Relaxation.

### SPECIAL NOTICE TO CORRESPONDENTS.

### Third Quarterly Word Competition, commenced July 4th, 1891, ends September 26th, 1891.

Competitors can enter for *all* quarterly competitions, but no competitor can take more than *one first prize* or *two* prizes of any kind during the year.

The word for dissection for this, the THIRTEENTH week of the quarter, being

"GRENADIER."

| Names. | Sept. 17th. | Totals. | Names. | Sept. 17th. | Totals. |
|---|---|---|---|---|---|
| Paignton | 10 | 354 | Christie | — | 44 |
| Psyche | — | — | Dulcamara | 10 | 449 |
| Hope | — | 47 | Nurse J. S. | 10 | 449 |
| Lightowlers | 10 | 488 | Qu'appelle | 10 | 488 |
| Wizard | — | 179 | E. M. S. | — | 68 |
| Wyameris | — | 46 | Jenny Wren | 10 | 444 |
| Dove | — | 46 | Carpe-diem | — | 65 |
| Punch | — | 181 | Grannie | — | 36 |
| Ivanhoe | — | — | Nurse G. P. | 4 | 276 |
| Tinie | — | 93 | Goodnight | — | 122 |
| Agamemnon | 10 | 470 | Gamp | — | 100 |
| Nurse Ellen | — | 86 | Charity | — | 104 |

**Notice to Correspondents.**

*Agamemnon* credited with 155 omitted.
*Qu'appelle* with 49 omitted.

### Fourth Quarterly Word Competition commences October 3rd, 1891.

All letters referring to this page which do not arrive at **140, Strand, London, W.C.**, by the *first post on Thursdays*, and are not addressed PRIZE EDITOR, will in future be disqualified and disregarded.

N.B.—Each paper must be signed by the author with his or her real name and address. A *nom de plume* may be added if the writer does not desire to be referred to by us by his real name. In the case of all prize-winners, however, the real name and address will be published.

## Figures and Tables

**Fig. 1. f1:**
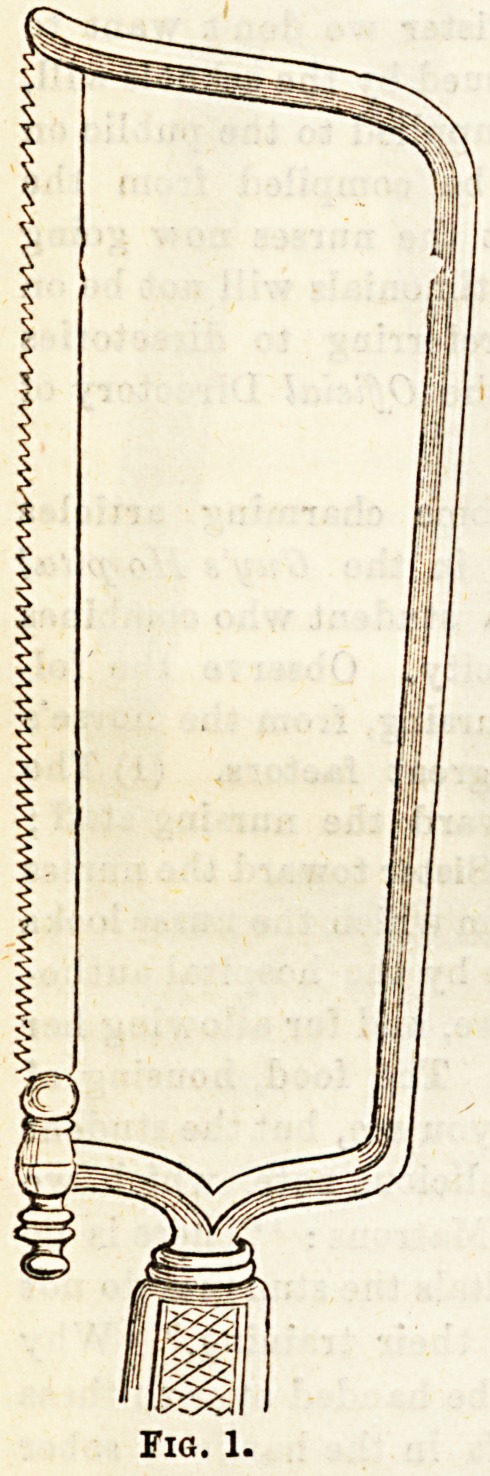


**Fig. 3. f2:**
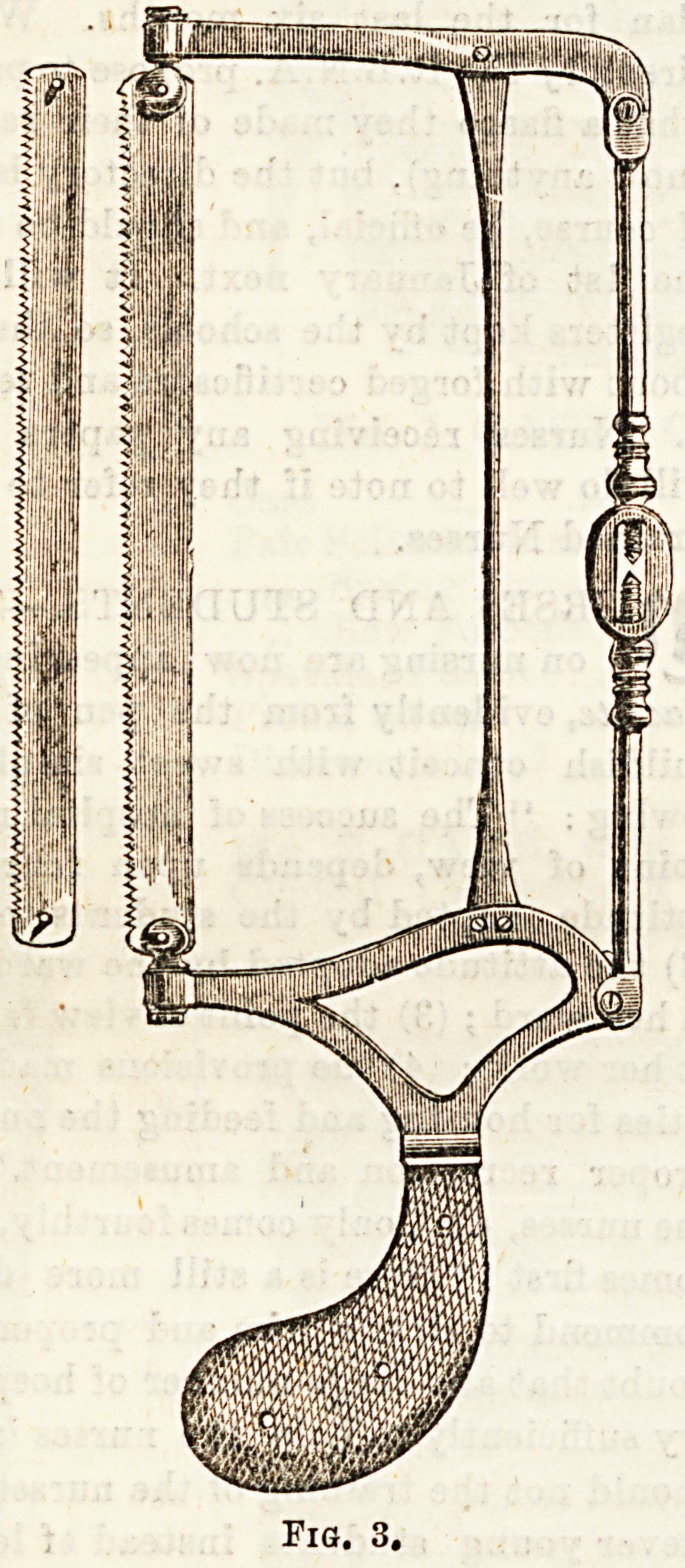


**Fig. 2. f3:**
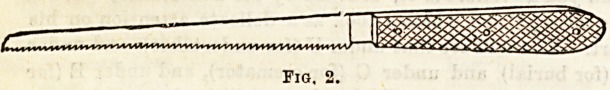


**Fig. 4. f4:**
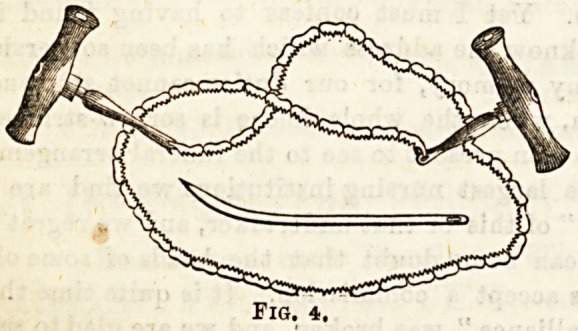


**Figure f5:**